# Absence of estrogen receptor beta leads to abnormal adipogenesis during early tendon healing by an up‐regulation of PPARγ signalling

**DOI:** 10.1111/jcmm.14604

**Published:** 2019-09-02

**Authors:** Xuting Bian, Tianyao Liu, Mei Zhou, Gang He, Yuanyuan Ma, Youxing Shi, Yunjiao Wang, Hong Tang, Xia Kang, Mingyu Yang, Jan‐Åke Gustafsson, Xiaotang Fan, Kanglai Tang

**Affiliations:** ^1^ Department of Orthopedic Surgery Southwest Hospital, Army Medical University Chongqing China; ^2^ Department of Developmental Neuropsychology, School of Psychology Third Military Medical University, Army Medical University Chongqing China; ^3^ Center for Nuclear Receptors and Cell Signaling University of Houston Houston TX; ^4^ Department of Biosciences and Nutrition, Center for Innovative Medicine Karolinska Institute Novum Sweden

**Keywords:** achilles tendon healing, estrogen receptor β, PPARγ signalling, tendon‐derived stem cells

## Abstract

Achilles tendon injury is one of the challenges of sports medicine, the aetiology of which remains unknown. For a long time, estrogen receptor β (ERβ) has been known as a regulating factor of the metabolism in many connective tissues, such as bone, muscle and cartilage, but little is known about its role in tendon. Recent studies have implicated ERβ as involved in the process of tendon healing. Tendon‐derived stem cells (TDSCs) are getting more and more attention in tendon physiological and pathological process. In this study, we investigated how ERβ played a role in Achilles tendon healing. Achilles tendon injury model was established to analyse how ERβ affected on healing process in vivo. Cell proliferation assay, Western blots, qRT‐PCR and immunocytochemistry were performed to investigate the effect of ERβ on TDSCs. Here, we showed that ERβ deletion in mice resulted in inferior gross appearance, histological scores and, most importantly, increased accumulation of adipocytes during the early tendon healing which involved activation of peroxisome proliferator‐activated receptor γ (PPARγ) signalling. Furthermore, in vitro results of ours confirmed that the abnormity might be the result of abnormal TDSC adipogenic differentiation which could be partially reversed by the treatment of ERβ agonist LY3201. These data revealed a role of ERβ in Achilles tendon healing for the first time, thereby providing a new target for clinical treatment of Achilles tendon injury.

## 1 BACKGROUND

Injury owing to sports has risen with the sports participation being increasingly popular in recent years, among which the Achilles tendon injury has been one of the most common. Achilles tendon injuries typically occur in the low vascularized region, 2 to 6 cm to the insertional point.^1^ In less than 10 years, the individual Achilles tendon injury rate increased from 2:100 000 to 12:100 000.^2^

According to our clinical observations and statistical data, one of the possible intrinsic factors that predispose athletes to Achilles tendon rupture is the level of oestrogen.^3^ The injury ratio of male to female ranges from 2:1 to 12:1.^4^ It was also reported that women athletes were more likely to suffer from tendon injury when the oestrogen level was low.^5^ And factors attributed to healing properties like proliferation rate and collagen I were lower in ovariectomized rats.^6^ However, a systematic review on pre‐ and post‐menopausal women showed that the effect of oestrogen supplementation on tendon was contradictory and inconsistent. But the review also suggested there was a need for further studies to understand the effects of female sex hormone supplementation on tendon tissue at a mechanical, morphological and molecular level.^7^ It is controversial for us to draw a conclusion about the relationship between oestrogen and tendon injury, which is possibly due to different properties of different tendon structures. Tendon possesses the capability to heal by a repair process controlled by tendon cells and their surrounding extracellular matrix (ECM).^8^ But damaged tendon heals slowly and rarely retains the structural integrity and mechanical strength of a healthy tendon.^9^, ^10^, ^11^ Recent evidence suggested that modulation of the early stage following tendon repair was important for improving healing.^12^ However, the precise mechanisms remain poorly understood.^13^ Thus, finding a new target for improving the healing of tendon injuries is one of the most essential challenges in sports medicine field.

ERβ is one kind of estrogen receptors belonging to nuclear transcription factors that are involved in the regulation of many complex physiological processes. For a long time, ERβ has been considered to be an important factor in regulating metabolic pathways and adipose tissue function.^14^ In recent days, modulation of ERβ has raised more attention, not only due to its promising role in more applications,^15^ such as some types of cancer and neuropathies, but also because it has less side effects compared to activating estrogen receptor α (ERα). As for tendon healing, one study on rat Achilles tendon showed that oestrogen has a positive effect.^6^

From a clinical perspective, ERβ‐selective agonists might be therapeutically useful. LY3201 is one of the special agonists of ERβ which has been proved to effectively and specifically activate ERβ but not ERα.^16^ Tendon possesses the capability to heal by a repair process controlled by tendon cells, especially TDSCs. TDSCs are multi‐potent adult stem cells with broad differentiation plasticity that makes them of great importance in cell‐based therapy for the repair of tendons.^17^ So, LY3201 and TDSCs were studied in our research for promising clinical application.

In the present study, we found that ERβ played an important role in the healing process of tendon injury. We showed that ERβ deletion in mice led to abnormal adipocyte accumulation via up‐regulation of expression of PPARγ signalling which might be related to TDSC adipogenesis that could be partially attenuated by LY3201.

## 2 MATERIALS AND METHODS

### 2.1 Animal model and surgical procedure

Male ERβ^−/−^ mice and their WT littermates were used in this study. The generation of the ERβ^−/−^ mice and their primary phenotype were described by Krege et al in 1998, which were all on a C57BL/6J background.^18^ Surgical procedures were performed as previously described by Palmes et al with 6‐month‐old mice that had reached maturity of skeletal system.^19^ In brief, (a) anaesthesia, (b) exposing both Achilles tendons from their origins from the gastrocnemius muscle down to their insertion into the calcaneum, (c) resecting the tendon of the plantaris muscle, (d) transecting the Achilles tendon by a standardized procedure at the position about 5 mm above its insertion into the calcaneum and about 5 mm below the first muscle fibres of the gastrocnemius, (e) re‐adapting the cut ends of the tendon with a Kirchmayr‐Kessler suture (6‐0 Dermalon) and further supported with a circular fine suture (10‐0 Dermalon), (f) closing the skin, (g) restricting the movement of the ankle by an external fixation designed by us in order to avoid suture failure due to overstretching of the operated tendons for the first two days after operation. After the operation, the mice were given eight days to repair corresponding to the early healing stage of injured tendons, after which the mice were killed. The whole hindlimb including the gastrocnemius‐Achilles tendon‐calcaneus complexes was retained for further processing.

### 2.2 Cell isolation, culture and reagents

Tendon‐derived stem cells were isolated as previously described by using 6‐week‐old male Sprague Dawley rats.^20^ Achilles tendon tissues were dissected with only the mid‐substance tissue collected and peritendinous connective tissue carefully removed. Then, the collective tissues were washed in 0.01M PBS and digested in 3 mg/mL type I collagenase (Sigma‐Aldrich) for 2 hours at 37°C. Then, the cells were filtered through 70 μm nylon mesh (Becton Dickinson) to yield a single‐cell suspension. After washing in 0.01 mol/L PBS, the released cells were centrifuged at 50 *g* for 5 minutes and resuspended in fresh culture media prepared by Dulbecco's modified Eagle's medium (DMEM) (Gibco) with 10% foetal bovine serum (FBS) and 1% penicillin/streptomycin (Pen/Strep) (all from Invitrogen, Carlsbad, CA). TDSCs were grown at 37°C and 5% CO_2_ and passaged when 70% confluent with the culture media changed every third day. Cells in passages 2‐3 were used for experiments. TDSCs were seeded onto 24‐well plates for cell staining and onto 6‐well plates for protein and RNA extraction. The identification of TDSCs is shown in Figure S3 according to Bi et al.^21^

The ERβ agonist LY3201 was a gift from Eli Lilly.^22^ The PPARγ agonist rosiglitazone (ROSI) was purchased from Selleck Chemicals (Houston, TX). All the agonists were dissolved with dimethyl sulfoxide (DMSO) purchased from Santa Cruz Biotechnology, Inc (Santa Cruz).

### 2.3 Histomorphometry and cellular morphometry

After fixing in 4% buffered formalin at 4°C for 24 hours followed by 30% sucrose at 4℃ for 24 hours, tendons were dehydrated and embedded in optimal cutting temperature compound (OCT) and processed for longitudinal sections (7 μm). Haematoxylin and eosin (HE) staining was used to examine the histology of Achilles tendon at defected zone and then graded by two blinded investigators to analyse the histological score modified by us based on histological scoring system of Stoll et al given in Table S1 according to Lin et al^23^ Oil Red O staining was performed to evaluate adipocyte accumulation in tendon scars. Immunohistochemistry and immunofluorescence were performed according to Bian et al.^24^ The sections were incubated in 3% H_2_O_2_ in phosphate‐buffered saline to quench endogenous peroxides for 20 minutes 37℃ (not needed in immunofluorescence) and then incubated in 0.3% Triton X‐100 in phosphate‐buffered saline for 30 minutes at 37℃. To block non‐specific binding, sections were incubated in 3% bovine serum albumin (BSA) for 30 minutes at 37℃. After that, they were incubated with primary antibodies against Ki67 (9106S1607D1, NeoMarkers), CD34 (ab81289, Abcam), Perilipin (ab61682, Abcam) and ERβ (PA1‐313, Thermo Fisher); all antibodies were diluted in 1% BSA and 0.1% Triton X‐100 for 2 hours at 37℃ and then overnight at 4°C, and negative controls using 1% BSA. Next day, after washing in 0.01 mol/L phosphate‐buffered saline (PBS), the sections were then incubated with biotin‐conjugated secondary antibodies or Cy3 (Donkey anti‐rabbit) secondary antibodies respectively for 2 hours at 37°C. To analyse apoptotic cell numbers, TUNEL assay was carried out according to the manufacturer's instructions (In Situ Cell Death Detection Kit, POD, Roche).

For cellular morphometry, after fixing in 4% buffered formalin for 30 minutes at room temperature (RT), TDSCs were washed in 0.01 mol/L PBS three times. Oil Red O staining was performed to evaluate adipocyte accumulation of tendon scars and adipogenic differentiation of TDSCs. For immunocytochemistry, TDSCs were incubated in 0.3% Triton X‐100 in phosphate‐buffered saline for 20 minutes at RT. To block non‐specific binding, sections were incubated in 3% BSA for 20 minutes at 37°C. After that, TDSCs were incubated with primary antibodies against PCNA (MAB424, Millipore), BrdU (555627, BD Pharmingen), PPARγ (#2443S, Cell Signaling Technology) and ERβ (PA1‐313, Thermo Fisher); all antibodies were diluted in 1% BSA and 0.1% Triton X‐100 overnight at 4°C, and negative controls using 1% BSA. Next day, after washing in 0.01M PBS, TDSCs were incubated with biotin‐conjugated secondary antibodies or Cy3 (Donkey anti‐rabbit) secondary antibodies respectively for 1.5 hours at RT.

All the stained sections and cells were viewed and photographed under a Zeiss (Oberkochen) Axiovert microscope equipped with a Zeiss AxioCam digital colour camera connected to the Zeiss AxioVision 3.0 system.

### 2.4 Quantitative reverse transcription polymerase chain reaction

Total RNA from tendon tissues and TDSCs was isolated with kit (Beyotime Institute of Biotechnology) and used for qRT‐PCR. Briefly, PCRs were pipetted on ice and each well contained 2.5 μL cDNA, 2.5 μL primer and 5 μL iTaq^TM^ Universal SYBR Green Supermix. Plates were subsequently sealed and centrifuged down for 20 seconds at 450 *g*, and then incubated at 95℃ for 5 minutes followed by 30 cycles of a three‐step temperature programme of 1 minutes at 95°C, 20 seconds at 65°C and 30 seconds at 72°C. The relative gene expression was quantified by densitometry and normalized to the amount of housekeeping gene glyceraldehyde 3‐phosphate dehydrogenase (GAPDH) and presented as fold change to WT controls. All PCR results were reproduced independently in five experiments. The primer sequences used in these experiments are listed in Table S2.

### 2.5 Western blot

Achilles tendons of both groups were isolated and homogenized in ice‐cold RIPA lysis buffer (Beyotime). After centrifugation of lysates (138000 *g*, 15 minutes, at 4°C), the protein concentration was determined via Bicinchoninic Acid Kit (Beyotime Institute of Biotechnology). Protein samples (20 μg/lane) were separated on a 12% SDS‐polyacrylamide gel for 50 minutes at 120 V and then transferred onto nitrocellulose membrane for 70 minutes at 120 V. Membranes were blocked with Tris‐buffered saline, containing 0.1% Tween‐20 and 5% fat‐free milk for 2 hours at RT. Membranes were then incubated overnight at 4°C with the rabbit antibodies against CD36 (ab133625, Abcam), PPARγ (#2443S, Cell Signaling Technology), FABP4 (ab216366, Abcam), Phos‐PTEN (sc‐101789, Santa Cruz), PTEN (sc‐7974, Santa Cruz), Phos‐AKT (#4060p, Cell Signaling Technology), AKT (#4685s, Cell Signaling Technology), p53 (sc‐47698, Santa Cruz), Phos‐ERK (sc‐7383, Santa Cruz), ERK (sc‐94, Santa Cruz), VEGFA (#9698s, Cell Signaling Technology), VEGFR (ab52917, Abcam) and GAPDH (KC‐5G5, Aksomics), followed by 2‐hours incubation at RT with a peroxidase‐conjugated goat anti‐rabbit immunoglobulin G (1:1000; Santa Cruz Biotechnology) and chicken anti‐mouse immunoglobulin G (1:1000; Santa Cruz Biotechnology). Finally, the NC membranes were scanned using the Odyssey Infrared Imaging System with the Odyssey Application software V1.2.15 for all antibodies. All Western blotting data were representative of at least five independent experiments.

### 2.6 Statistical analysis

Statistical analysis was performed with SPSS 25.0 software (SPSS Inc). Statistical differences between two groups were determined using two‐tailed unpaired Student's *t* test, or two‐tailed non‐parametric Mann‐Whitney test, or one‐way analysis of variance followed by Fisher's protected least significant difference post hoc test among three or five groups. Sample size and experimental reproduction are indicated for each method. Results are presented as mean ± SD Differences reached statistical significance at *P < .05, **P < .01, ***P < .001.

## 3 RESULTS

### 3.1 ERβ^−/−^ tendon scars have inferior gross appearance, lower histological scores and cell density paralleled with more increased adipocytes and vessel accumulation

**Figure 1:**
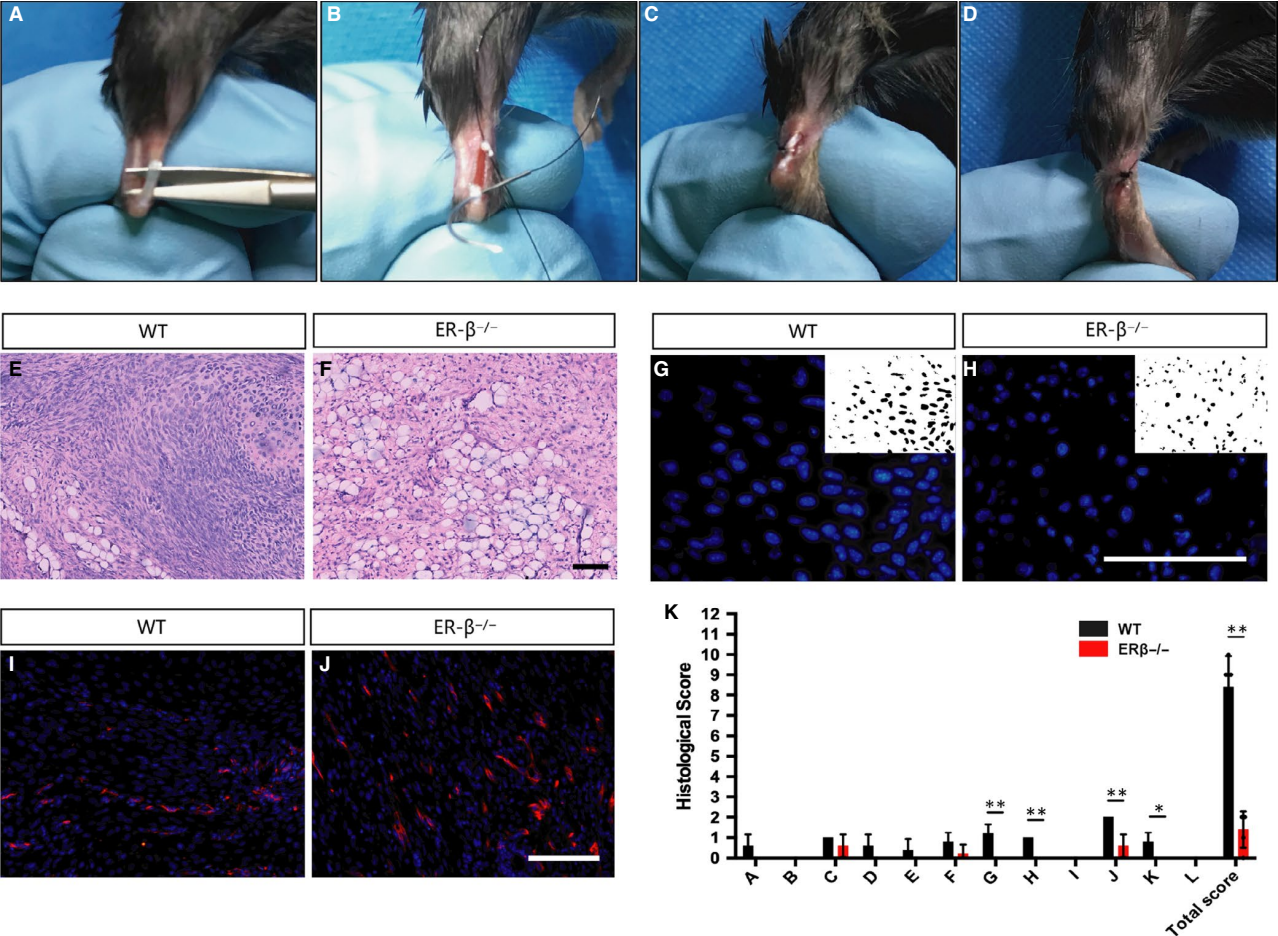
ERβ^−/−^ tendon scars have an inferior tendon repair process. A‐D, Surgery process of mouse Achilles tendon. E, F Low‐magnification HE staining indicates a very different scar organization with clear adipocyte accumulation in ERβ^−/−^ mice. (G,H) Cell density in the healing region was significantly lower in ERβ^−/−^ mice compared with WT controls. (I,J) Number of CD34‐labelled blood vessels was significantly higher in ERβ^−/−^ mice compared with WT controls. K, Evaluation of tendon healing using an established histological scoring system revealed that ERβ^−/−^ mice had a significantly lower histological score compared with WT controls. Data are represented as mean ± SEM (n = 5), *P < .05, **P < .01. Scale Bars: 100μm

**Figure 2:**
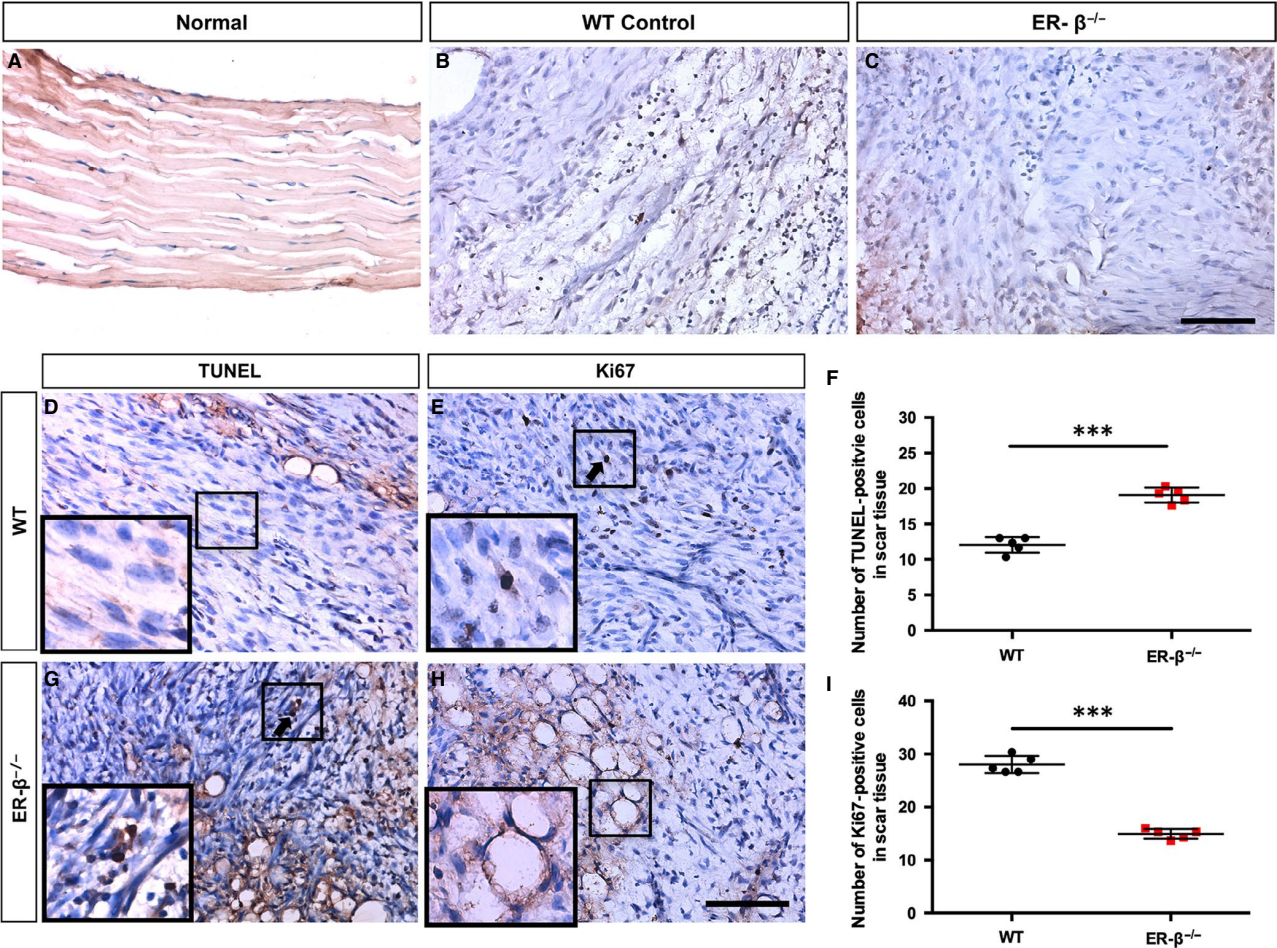
ERβ^−/−^ tendon scars have reduced cell proliferation, whilst increased cell apoptosis. A‐C, Immunohistochemistry of ERβ revealed a little expression in normal mouse Achilles tendon (A), more expression in early healing of WT mouse Achilles tendon (B) and no expression in ER‐β^−/−^ mouse injured Achilles tendon. ERβ^−/−^ mouse Achilles tendon (C). (D,G,F) TUNEL staining and statistical analysis showed more apoptotic cells in ERβ^−/−^ tendon scars. (E,H,I) Immunohistochemistry of Ki67 and statistical analysis revealed decreased cell proliferation in ERβ^−/−^ tendon scars. Data are represented as mean ± SEM (n = 5), ***P < .001. Scale Bars: 100μm

To analyse ERβ involvement during early tendon healing, we established a mouse model of full‐thickness Achilles tendon injury (Figure 1A‐D). We analyzed injured tendons in the eighth postoperative day, a time‐point of repair process characterized by scar formation, inflammatory cell invasion and high cell proliferation. First, we carried out immunohistochemistry of ERβ, the result showed more ERβ‐expressing cells in injured tendons compared with normal ones, and there was no ERβ expression in ERβ^−/−^ mice (Figure 2A‐C). Haematoxylin and eosin (HE) staining of sectioned tendons revealed a very different scar organization in ERβ^−/−^ mice, as indicated by significantly inferior total histological scores compared with WT littermates (Figure 1E,F,K). Quantitatively, total cell density was significantly lower in the ERβ^−/−^ mice at eight post‐operative days (Figure 1G,H, Figure S1B). At the same time, the mean area of adipocyte accumulation (Figure 1E,F), the number of blood vessels observed in HE staining analyses and validated by immunofluorescence staining, and quantification of CD34‐positive areas were significantly higher in the scar sites of ERβ^−/−^ mice compared with WT control (Figure 1I,J, Figure S1A). The above data revealed for the first time that the absence of ERβ leads to an inferior morphological outcome and lower cellular density, whilst it activates adipocyte accumulation as well as vessel numbers in the early repair region of injured tendons.

### 3.2 ERβ^−/−^ tendons have less cell proliferation, whilst more cell apoptosis

To test whether the reduction in cell numbers was due to a decreased proliferation or increased apoptosis, we carried out proliferative and apoptotic assays by immunohistochemistry of Ki67 and terminal deoxynucleotidyl transferase‐mediated dUTP‐biotin nick end labelling (TUNEL) staining (Figure 2D,E,G,H). TUNEL assays showed that ERβ^−/−^ scars had a higher number of apoptotic cells (Figure 2F). Furthermore, Ki67 analysis confirmed a lower number of proliferating cells at the scar site of injured Achilles tendon in ERβ^−/−^ mice than WT controls (Figure 2I). The above data demonstrated that the absence of ERβ results in reduced cell proliferation and increased cell apoptosis of tendon scars during early healing process.

### 3.3 ERβ^−/−^ tendon scars have more adipocyte accumulation and up‐regulation of PPARγ signalling pathway

**Figure 3:**
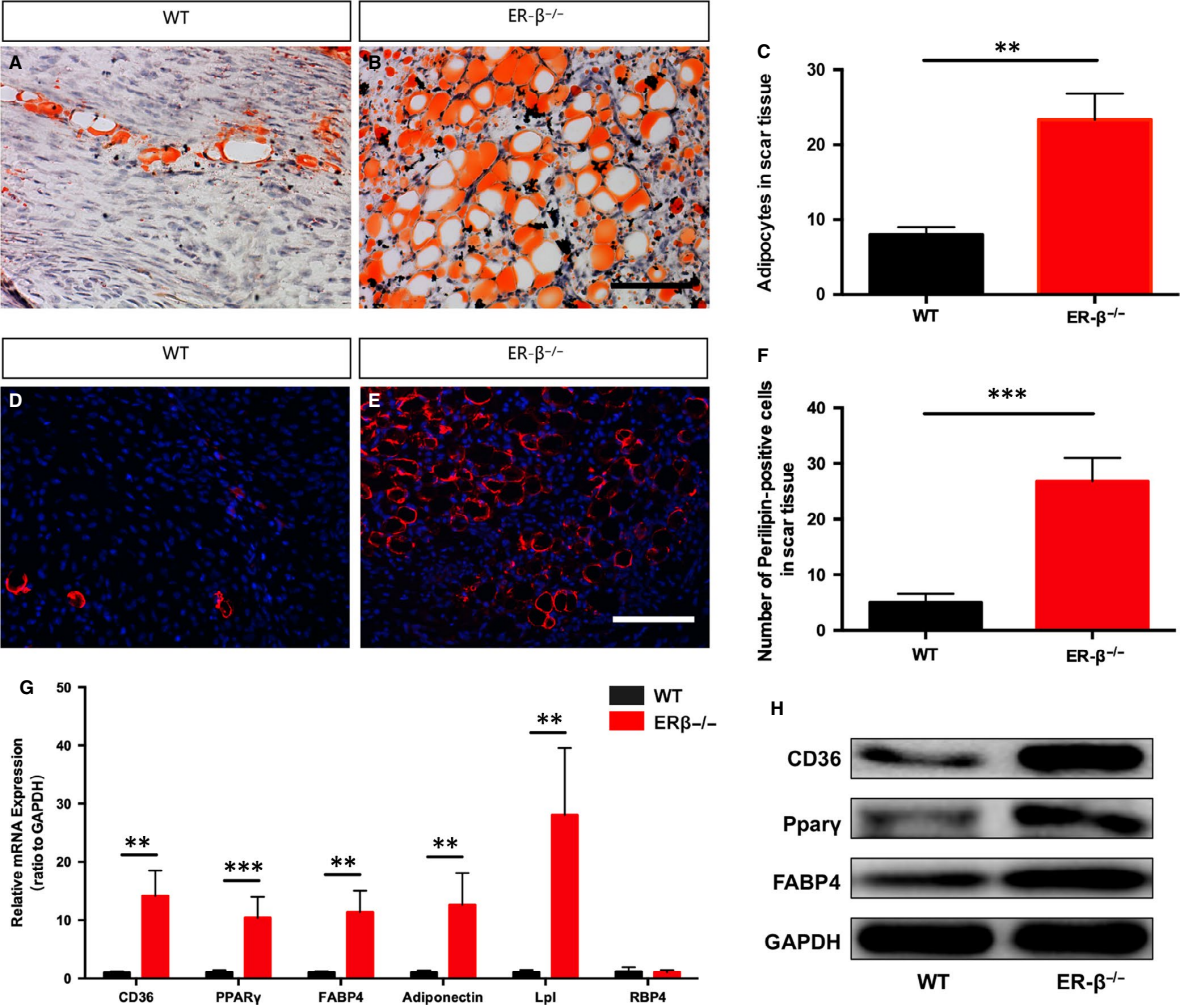
ERβ deficiency results in more adipocyte accumulation and activation of PPARγ signalling pathway. (A,B) Oil Red O staining represented adipocytes in tendon scar region. C, Adipocytes labelled by Oil Red O staining were significantly increased in ERβ^−/−^ mice compared with WT controls. (D,E) Immunofluorescence of CD34‐positive adipocytes in tendon scar region. F, CD34‐positive adipocytes were significantly increased in ERβ^−/−^ mice compared with WT control. G, ERβ^−/−^ mice tendon scars displayed significantly higher expression levels of CD36, PPARγ, FABP4, adiponectin and Lpl, but no difference compared with WT controls. H, Representative immunoblots of PPARγ signalling pathway including CD36, PPARγ and FABP4 were significantly activated in ERβ^−/−^ mice tendon scars compared with WT control. Data are represented as mean ± SEM (n = 5), **P < .01, ***P < .001. Scale Bars: 100 μm

In order to further investigate the effect of the absence of ERβ on adipogenesis during early tendon healing, we carried out Oil Red O staining (Figure 3A,B), and the result showed that there was more adipocyte accumulation in the ERβ^−/−^ mice compared with the WT controls (Figure 3C). The same result could be further confirmed by the immunofluorescence of Perilipin (Figure 3D,E,F). PPARγ signalling pathway plays a key role in regulating adipogenesis. To investigate PPARγ signalling involvement in vivo, we examined PPARγ activity and PPARγ target genes in ERβ^−/−^ mice and their WT controls. The results showed that adipose mRNA expression of PPARγ target genes involving CD36, FABP4, adiponectin and lipoprotein lipase (Lpl) was significantly up‐regulated in ERβ^−/−^ mice, but RBP4 was not affected (Figure 3G). Furthermore, Western blot results of adipogenesis‐related protein expression including CD36, PPARγ and FABP4, which are involved in PPARγ signalling pathway, revealed significant increase in the ERβ^−/−^ mice compared with the WT controls (Figure 3H).

### 3.4 Dose‐dependent LY3201 activation of ERβ promotes the proliferative capacity and inhibits the adipogenic differentiation of TDSCs

**Figure 4:**
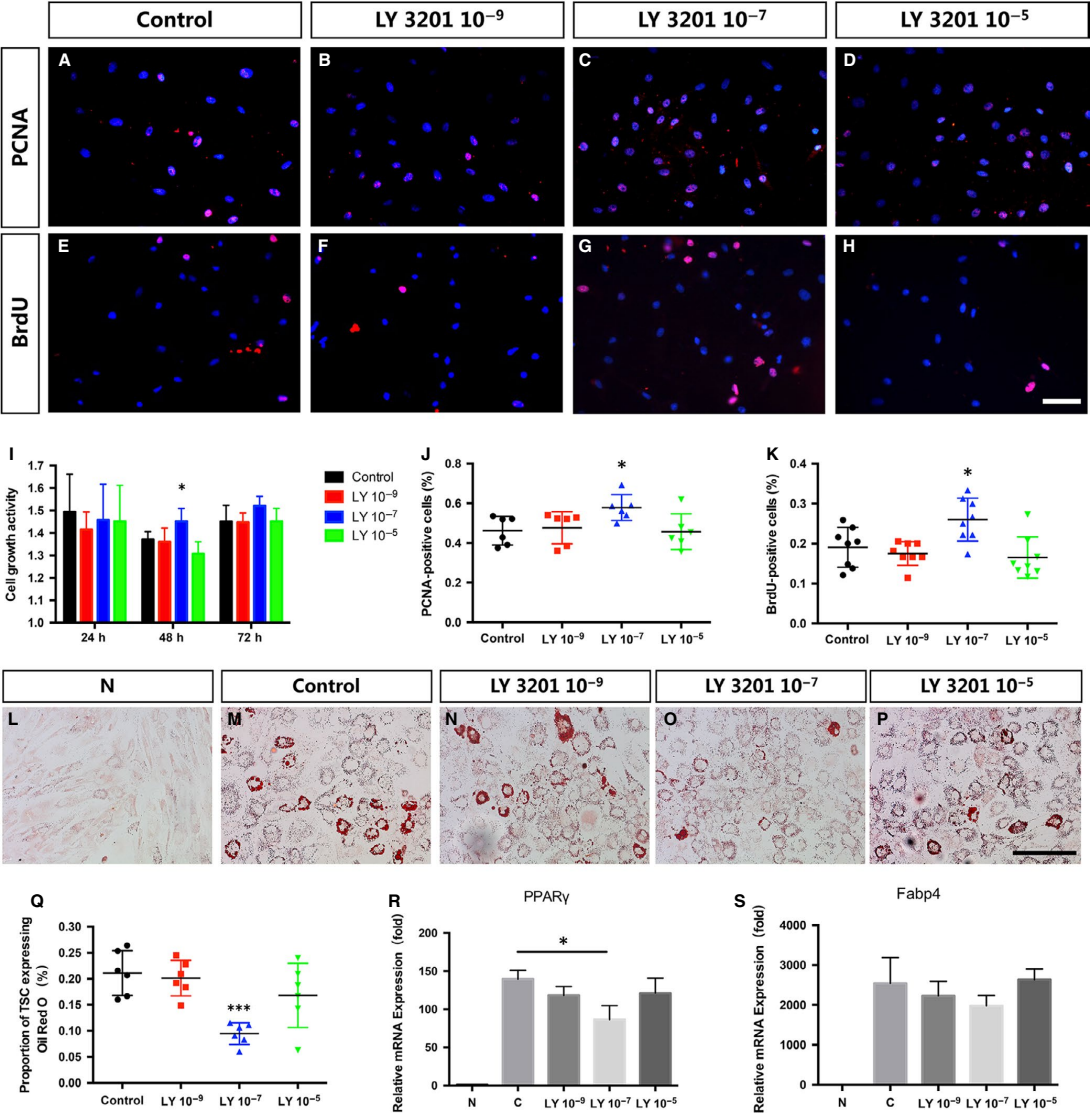
Activation of ERβ induces the proliferative capacity and inhibits the adipogenic differentiation of TDSCs. (A‐D,J) Immunofluorescence of PCNA and statistic analysis revealed more numbers of PCNA‐positive TSCs with the treatment of LY3201 at the dose of 1 × 10^−7^ mol/L versus control, but no difference with other concentrations. (E‐H,K) Immunofluorescence of BrdU and statistic analysis revealed more numbers of BrdU‐positive TSCs with the treatment of LY3201 at the dose of 1 × 10^−7^ mol/L versus control, but no difference with other concentrations. I, CCK‐8 assay revealed the treatment of LY3201 at the dose of 1 × 10^−7^ mol/L for 48 h promoted proliferation of TSCs, but no difference with other concentrations for other periods. (L‐Q) Oil Red O staining and statistic analysis revealed inhibition of adipogenic differentiation of TDSCs by 1 × 10^−7^ mol/L LY3201, but no significant difference with other concentrations. (R,S) qRT‐PCR revealed down‐regulated mRNA levels of PPARγ and FABP4 by 1 × 10^−7^ mol/L LY3201, but no significant difference with other concentrations. Data are represented as mean ± SEM (n = 5), *P < .05, ***P < .001. Scale Bars: 100 μm

In order to investigate whether the proliferative capacity of TDSCs was affected by activation of ERβ, cells were cultured with different concentration of LY3201 for 24, 48 and 72 hours with DMEM culture media. CCK‐8 assay revealed the treatment of LY3201 at the dose of 1 × 10^−7^ mol/L for 48 hours promoted proliferation of TSCs, but no difference with other concentrations for other periods (Figure 4I). The same result was further confirmed by immunofluorescence of both PCNA and BrdU (Figure 4A‐H,J,K). Then, in order to confirm the effect of ERβ on TDSCs’ adipogenic differentiation further, TDSCs were treated with different concentrations of LY3201 added into phenol red‐free adipogenic medium for 14 days (Figure 4L‐P). Our results showed that adipogenic differentiation of TDSCs was inhibited by LY3201 at the concentration of 1 × 10^−7^ mol/L, but the same effect could not be found either at the concentration of 1 × 10^−5^ mol/L or 1 × 10^−9^ mol/L (Figure 4Q). And genes related to adipogenic differentiation, PPARγ and FABP4, also presented the same characteristics (Figure 4R,S). These results showed that activation of ERβ by LY3201 promoted the proliferative capacity and inhibited the adipogenic differentiation of TDSCs, and this kind of effect was dose‐dependent.

### 3.5 Dose‐dependent LY3201 activation of ERβ inhibits the expression of PPARγ and adipogenic differentiation of TDSCs

**Figure 5:**
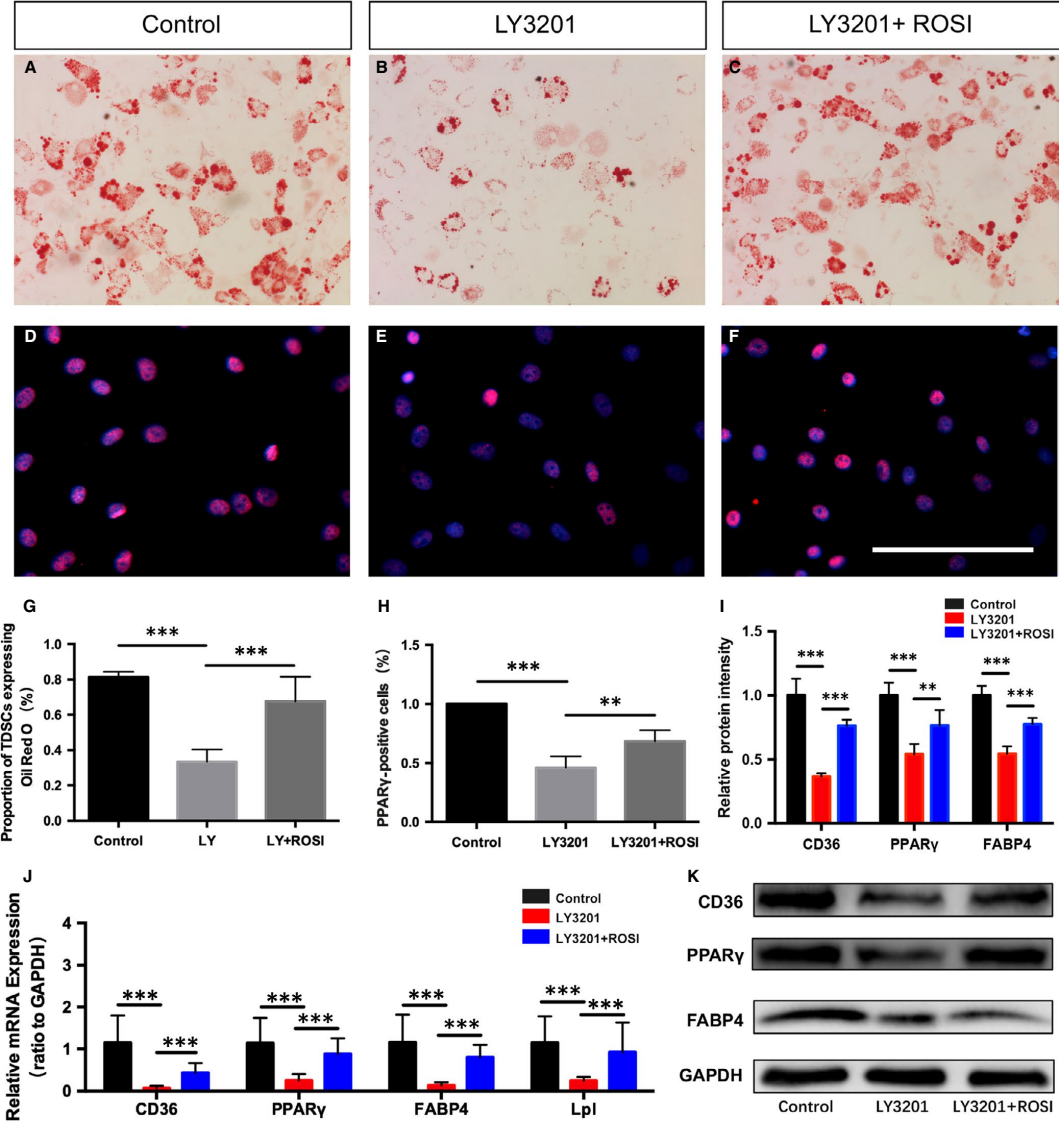
Activation of ERβ inhibits adipogenic differentiation of TDSCs by a negative crosstalk with PPARγ signalling. (A‐C,G) Oil Red O staining and statistic analysis revealed inhibition of adipogenic differentiation of TDSCs by 1 × 10^−7^ mol/L LY3201, which could be partly re‐activated by the treatment of ROSI. (D‐F,H) Immunofluorescence of PPARγ and statistic analysis showed a consistent change with Oil Red O staining result. J qRT‐PCR revealed the change of mRNA levels related to PPARγ signalling including CD36, PPARγ, FABP4 and Lpl with the treatment of LY3201 and ROSI. (I,K) Representative immunoblots and statistic analysis of PPARγ signalling pathway including CD36, PPARγ and FABP4 with the treatment of LY3201 and ROSI. Data are represented as mean ± SEM (n = 5), **P < .01, ***P < .001. Scale Bars: 100 μm

In order to further investigate whether PPARγ signalling pathway was involved in the process of LY3201 inhibiting adipogenic differentiation of TDSCs, we continued to carry out adipogenic differentiation culture to TDSCs with ERβ agonist LY3201 and PPARγ antagonist ROSI. The results showed that the inhibition by LY3201 of adipogenic differentiation of TDSCs was reversed with the treatment of 10μM ROSI (Figure 5A‐C,G). Next, immunofluorescence of PPARγ showed a significant decrease by LY3201 and an increase again with the treatment of ROSI (Figure 5D‐F,H). Finally, we examined related genes of PPARγ signalling pathway, both PCR and WB results confirmed the involvement (Figure 5I,J,K), and the results showed a significant activation of it.

## 4 DISCUSSION

Tendon injury has been a big challenge affecting patients by the failed healing and relapse, especially in athletes.^25^ It is reported that oestrogen may be related to the occurrence of tendon injury,^5^, ^26^ and there have also been many studies investigating the effects of oestrogen on tendon injury in post‐menstrual women or ovariectomized animals,^6^, ^26^, ^27^ which had the limitations of interference of age (post‐menstrual women) and inaccurate target (ovariectomized animals), respectively. In many other studies, investigations have been conducted especially with regard to possible treatments of diseases in tendons by the means of hormone replacement therapy.^28^, ^29^ However, only a few studies committed themselves to an approach at a molecular level, especially data by using a knockout model.

The natural healing process in tendon is considered to consist of three overlapping phases: inflammation, proliferation and matrix remodelling.^30^ Early tendon healing process mainly contains proliferation of fibroblasts, inflammatory cells and stem cells such as bone marrow‐derived stem cells (BMSCs) and TDSCs, which is of great importance creating conditions for next step of healing.^31^, ^32^ Therefore, in our study ERβ^−/−^ mice were used to establish a full‐thickness Achilles tendon injury for further exploration of the potential role of ERβ in the early healing process. Based on all the results, we demonstrated for the first time that there was more expression of ERβ in injured Achilles tendon, and the absence of ERβ in mouse Achilles tendon led to poor early healing process characterized by increased angiogenesis, reduced cell proliferation, increased cell apoptosis and adipocyte accumulation.

Angiogenesis during early inflammation stage is related to tendon healing.^33^ General speaking, an acceleration of angiogenesis regulates inflammation reaction and healing process.^34^ The importance of PPARγ in angiogenesis was demonstrated by the generation of knockout animals in 1999.^35^ In our study, we found that there were significantly increased vessel numbers labelled by CD34 in ERβ knockout mice and significant activation of PPARγ. To investigate how PPARγ affected it, VEGFA and VEGFR were assessed by Western blot. The results showed an increased expression of VEGFA and VEGFR in ERβ knockout mice (Figure S5A,B,C). It has been reported PPARγ played a role in up‐regulating VEGFA production and release in mature adipocytes.^36^ Thus, the mechanism for increased CD34‐labelled blood vessels includes enhancement of VEGFA and VEGFR by the activation of PPARγ.

DAPI revealed a decreased cell density in ERβ knockout mice, and further results of TUNEL staining and immunohistochemistry of Ki67 confirmed there were more cell apoptosis, whilst less cell proliferation. It has been reported that PPARγ/PTEN/AKT pathway played a role in the apoptosis of differentiated human embryonic stem cells.^37^, ^38^, ^39^ In order to confirm whether it is involved in healing process of ERβ knockout mice tendon, we examined proteins related to this signalling pathway. The Western blot results showed that there was a up‐regulation of both PTEN and phosphate‐PTEN, and then played a negative role in the AKT signalling pathway (Figure S6A‐E), and finally an increased p53 expression in ERβ knockout mice (Figure S6F). Thus, the absence of ERβ during early tendon healing induced apoptosis through the PPARγ/PTEN/AKT signalling pathway. At the same time, we also examined expression of phosphate‐ERK and ERK, which has been demonstrated to be related to cell proliferation.^40^, ^41^ The results showed a significant decrease of phosphate‐ERK in ERβ knockout mice tendon with no change of total ERK, which confirmed the involvement of ERK (Figure S6A,G,H). And in vitro results confirmed enhanced proliferative capacity of TDSCs with the treatment of ERβ agonist LY3201. But the mechanism of how ERβ modulates this process was not researched in this study.

Perfect re‐establishment of injured tendon is supposed to recover well‐organized collagen structure; both abnormal ectopic endochondral ossification and adipocyte accumulation will interrupt natural healing process. Adipocyte accumulation is one of the common pathological changes that occur in ruptured tendons.^23^, ^42^ Our study found that the absence of ERβ resulted in adipocyte accumulation in the scar site of injured Achilles tendon. It was reported that PPARγ was down‐regulated by the activation of estrogen receptors in hepatocellular carcinoma.^43^ Another previous research has also shown that blockade of PPARγ signalling in adipose tissue of ER‐β^−/−^ mice resulted in a reversal of the metabolic phenotype corroborating the importance of adipose PPARγ in the high‐fat diet model.^44^ There are complex interactions between PPARγ and ERβ.^45^, ^46^, ^47^ Interestingly, in our study, we found that ER‐β^−/−^ mice had more adipocyte accumulation revealed by HE staining, Oil Red O staining and immunofluorescence of Perilipin compared with WT controls. At the same time, we found significant increase in CD36, PPARγ and FABP4 that were all involved in PPARγ signalling pathway regulating adipocyte differentiation both at RNA and at protein levels. All these results in vivo brought up a hint that the absence of ERβ leads to adipocyte accumulation through the activation of PPARγ signalling pathway.

In order to confirm this further, TDSCs were tested in vitro. Recent data showed that TDSCs showed higher clonogenicity, proliferation and multi‐lineage differentiation potential compared with BMSCs in vitro.^21^, ^48^ LY 3201 is one of the specific agonists of ERβ that has been proved to effectively and characteristically activate ERβ but not α that renders it of great clinical application value.^16^ Consistent with the in vivo findings, our in vitro results showed that activation of ERβ by agonist LY3201 inhibited adipogenic differentiation of TDSCs after culture in adipogenic medium for 14 days through PPARγ signalling pathway. To directly demonstrate the role of PPARγ during adipogenic differentiation of TDSCs with the absence of ERβ, PPARγ signalling was activated by PPARγ agonist ROSI again. The results showed that activation of PPARγ by ROSI reversed the effects of LY3201. TDSCs could differentiate into osteogenic, chondrogenic and adipogenic lineages so that they could form tendon‐like, cartilage‐like, adipose‐like tissue.^21^, ^49^ Thus, on the one hand, ERβ absence leads to more adipocyte differentiation during early healing process which interrupts ideal repair. On the other hand, whether increased adipocyte differentiation inhibits tenocyte differentiation needs further investigation.

## 5 CONCLUSIONS

**Figure 6:**
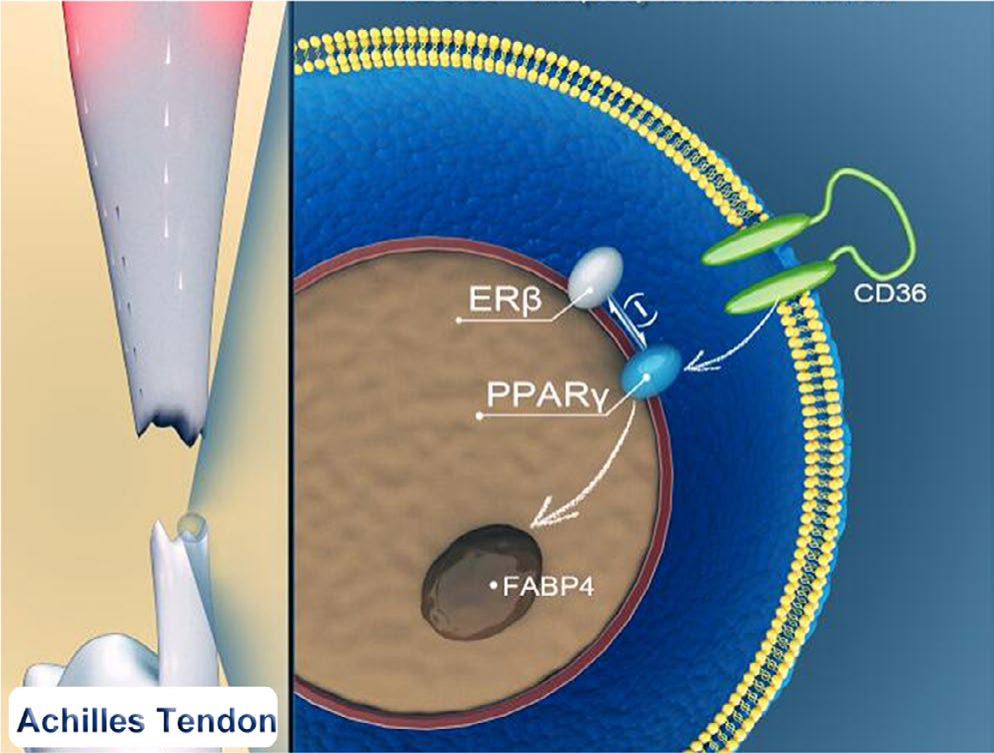
Model of adipogenesis action of ERβ is mediated by a negative crosstalk with PPARγ. During early healing process of tendon injury, absence of ERβ will augment the PPARγ expression because of the negative crosstalk between the two so that PPARγ signalling is activated including upstream CD36 and target FABP4, the result is more TDSCs differentiating into adipocytes leads to a poor repair

Collectively, our present findings reveal for the first time the absence of ERβ leads to an abnormal healing process mainly characterized by more adipocyte accumulation during early repair of mouse Achilles tendon which involves an augmented PPARγ signalling. Furthermore, in vitro results confirm that the abnormity might be due to abnormal TDSC adipogenic differentiation which could be partially reversed by the treatment of ERβ agonist (Figure 6). Implications of these findings might involve a new target for clinical treatment of Achilles tendon injury.

## CONFLICT OF INTEREST

The authors declare that they have no conflict of interests.

## AUTHORS’ CONTRIBUTIONS

XTB, MZ, GH, YXS, YJW and HT performed experiments. XTB, XK, XTF and KLT conceived the ideas and designed the work. TYL and YYM provided animal care. MYY provided important advice on data analysis. All authors read and approved the final manuscript.

## Data Availability

All data generated or analysed during this study are included in this published article.

## References

[1] Muller SA , Evans CH , Heisterbach PE , Majewski M . The Role of the Paratenon in Achilles Tendon Healing: A Study in Rats. Am J Sports Med. 2018;46:1214‐1219. 29505741 10.1177/0363546518756093

[2] Hess GW . Achilles tendon rupture: a review of etiology, population, anatomy, risk factors, and injury prevention. Foot Ankle Spec. 2010;3:29‐32. 20400437 10.1177/1938640009355191

[3] Leblanc DR , Schneider M , Angele P , Vollmer G , Docheva D . The effect of estrogen on tendon and ligament metabolism and function. J Steroid Biochem Mol Biol. 2017;172:106‐116. 28629994 10.1016/j.jsbmb.2017.06.008

[4] So V , Pollard H Management of Achilles tendon disorders. A case review. Australas Chiropr Osteopathy. 1997;6:58‐62. 17987151 PMC2050628

[5] Möller‐nielsen J , Hammar M . Women's soccer injuries in relation to the menstrual cycle and oral contraceptive use. Med Sci Sports Exerc. 1989;21:126‐129. 2709976

[6] Torricelli P , Veronesi F , Pagani S , et al. In vitro tenocyte metabolism in aging and oestrogen deficiency. Age (Dordr). 2013;35:2125‐2136. 23274854 10.1007/s11357-012-9500-0 PMC3825001

[7] Ganderton C , Semciw A , Cook J , Pizzari T . The effect of female sex hormone supplementation on tendon in pre and postmenopausal women: a systematic review. J Musculoskelet Neuronal Interact. 2016;16: 92‐104. 27282453 PMC5114352

[8] Wu F , Nerlich M , Docheva D . Tendon injuries: basic science and new repair proposals. EFORT Open Rev. 2017;2:332‐342. 28828182 10.1302/2058-5241.2.160075 PMC5549180

[9] Soma CA . Mandelbaum BR . Achilles tendon disorders. Clin Sports Med. 1994;13:811‐823. 7805108

[10] Asplund CA , Best TM . Achilles tendon disorders. BMJ. 2013;346:f1262. 23482943 10.1136/bmj.f1262

[11] Singh D . Acute Achilles tendon rupture. BMJ. 2015;351:h4722. 26494035 10.1136/bmj.h4722

[12] Thomopoulos S , Parks WC , Rifkin DB , Derwin KA . Mechanisms of tendon injury and repair. J Orthop Res. 2015;33:832‐839. 25641114 10.1002/jor.22806 PMC4418182

[13] Shapiro E , Grande D , Drakos M . Biologics in Achilles tendon healing and repair: a review. Curr Rev Musculoskelet Med. 2015;8:9‐17. 25655258 10.1007/s12178-015-9257-z PMC4596191

[14] Ropero A , Alonsomagdalena P , Quesada I , Nadal A . The role of estrogen receptors in the control of energy and glucose homeostasis. Steroids. 2008;73:874‐879. 18249429 10.1016/j.steroids.2007.12.018

[15] Tu J , Jufri NF . Estrogen signaling through estrogen receptor beta and G‐protein‐coupled estrogen receptor 1 in human cerebral vascular endothelial cells: implications for cerebral aneurysms. Biomed Res Int. 2013;2013:524324. 24319683 10.1155/2013/524324 PMC3844273

[16] Miao YF , Su W , Dai YB , et al. An ERbeta agonist induces browning of subcutaneous abdominal fat pad in obese female mice. Sci Rep. 2016;6:38579. 27922125 10.1038/srep38579 PMC5138613

[17] Lui PP . Identity of tendon stem cells–how much do we know? J Cell Mol Med. 2013;17:55‐64. 23279609 10.1111/jcmm.12007 PMC3823136

[18] Krege JH , Hodgin JB , Couse JF , et al. Generation and reproductive phenotypes of mice lacking estrogen receptor beta. Proc Natl Acad Sci U S A. 1998;95:15677‐15682. 9861029 10.1073/pnas.95.26.15677 PMC28103

[19] Palmes D , Spiegel HU , Schneider TO , et al. Achilles tendon healing: long‐term biomechanical effects of postoperative mobilization and immobilization in a new mouse model. J Orthop Res. 2002;20:939‐946. 12382957 10.1016/S0736-0266(02)00032-3

[20] Chen W , Tang H , Zhou M , Hu C , Zhang J , Tang K . Dexamethasone inhibits the differentiation of rat tendon stem cells into tenocytes by targeting the scleraxis gene. J Steroid Biochem Mol Biol. 2015;152:16‐24. 25906952 10.1016/j.jsbmb.2015.04.010

[21] Bi Y , Ehirchiou D , Kilts TM , et al. Identification of tendon stem/progenitor cells and the role of the extracellular matrix in their niche. Nat Med. 2007;13:1219‐1227. 17828274 10.1038/nm1630

[22] Norman BH , Richardson TI , Dodge JA , et al. Bioorganic RKJ, letters mc. Benzopyrans as selective estrogen receptor beta agonists (SERBAs). Part 4: functionalization of the benzopyran A‐ring. Bioorg Med Chem Lett. 2007;17:5082‐5085. 17662603 10.1016/j.bmcl.2007.07.009

[23] Lin D , Alberton P , Caceres MD , Volkmer E , Schieker M , Docheva D . Tenomodulin is essential for prevention of adipocyte accumulation and fibrovascular scar formation during early tendon healing. Cell Death Dis. 2017;8:e3116. 29022912 10.1038/cddis.2017.510 PMC5682675

[24] Bian X , Zhong H , Li F , et al. LXR agonist rescued the deficit in the proliferation of the cerebellar granule cells induced by dexamethasone. Biochem Biophys Res Commun. 2016;477:826‐833. 27369072 10.1016/j.bbrc.2016.06.142

[25] Sharma P , Maffulli N . Tendon injury and tendinopathy: healing and repair. J Bone Joint Surg Am. 2005;87(1):187‐202. 15634833 10.2106/JBJS.D.01850

[26] Wojtys EM , Huston LJ , Boynton MD , Spindler KP , Lindenfeld TN . The effect of the menstrual cycle on anterior cruciate ligament injuries in women as determined by hormone levels. Am J Sports Med. 2002;30:182‐188. 11912085 10.1177/03635465020300020601

[27] Circi E , Akpinar S , Balcik C , et al. Biomechanical and histological comparison of the influence of oestrogen deficient state on tendon healing potential in rats. Int Orthop. 2009;33:1461‐1466. 19387642 10.1007/s00264-009-0778-1 PMC2899125

[28] Ewies A . Changes in extracellular matrix proteins in the cardinal ligaments of post‐menopausal women with or without prolapse: a computerized immunohistomorphometric analysis. Hum Reprod. 2003;18:2189‐2195. 14507843 10.1093/humrep/deg420

[29] Ewies AA , Elshafie M , Li J , et al. Changes in transcription profile and cytoskeleton morphology in pelvic ligament fibroblasts in response to stretch: the effects of estradiol and levormeloxifene. Mol Hum Reprod. 2008;14:127‐135. 18184756 10.1093/molehr/gam090

[30] Docheva D , Müller SA , Majewski M , Evans CH . Biologics for tendon repair. Adv Drug Deliv Rev. 2015;84:222‐239. 25446135 10.1016/j.addr.2014.11.015 PMC4519231

[31] Tan Q , Lui PP , Lee YW . In vivo identity of tendon stem cells and the roles of stem cells in tendon healing. Stem Cells Dev. 2013;22:3128‐3140. 23815595 10.1089/scd.2013.0073 PMC3857013

[32] Linderman SW , Gelberman RH , Thomopoulos S , Shen H . Cell and biologic‐based treatment of flexor tendon injuries. Oper Tech Orthop. 2016;26:206‐215. 28042226 10.1053/j.oto.2016.06.011 PMC5193226

[33] Chamberlain CS , Crowley E , Vanderby R . The spatio‐temporal dynamics of ligament healing. Wound Repair Regen. 2009;17:206‐215. 19320889 10.1111/j.1524-475X.2009.00465.x PMC3214965

[34] Sun K , Huang R , Wu S , et al. The pleiotropic effects of PPARs on vascular cells and angiogenesis: implications for tissue engineering. Curr Stem Cell Res Ther. 2016;11:265‐273. 26951130 10.2174/1574888x1103160303181155

[35] Kotlinowski J , Jozkowicz A . PPAR gamma and angiogenesis: endothelial cells perspective. J Diabetes Res. 2016;2016:8492353. 28053991 10.1155/2016/8492353 PMC5174176

[36] Hasan AU , Ohmori K , Konishi K , et al. Eicosapentaenoic acid upregulates VEGF‐A through both GPR120 and PPARgamma mediated pathways in 3T3‐L1 adipocytes. Mol Cell Endocrinol. 2015;406:10‐18. 25697344 10.1016/j.mce.2015.02.012

[37] Fang H , Fang W , Cao H , et al. Di‐(2‐ethylhexyl)‐phthalate induces apoptosis via the PPARgamma/PTEN/AKT pathway in differentiated human embryonic stem cells. Food Chem Toxicol. 2019;131:110552. 31163220 10.1016/j.fct.2019.05.060

[38] Wang XM , Yao M , Liu SX , Hao J , Liu QJ , Gao F . Interplay between the Notch and PI3K/Akt pathways in high glucose‐induced podocyte apoptosis. Am J Physiol Renal Physiol. 2014;306:F205‐F213. 24226527 10.1152/ajprenal.90005.2013

[39] Li Y , Xia J , Jiang N , et al. Corin protects H2O2‐induced apoptosis through PI3K/AKT and NF‐kappaB pathway in cardiomyocytes. Biomed Pharmacother. 2018;97:594‐599. 29101802 10.1016/j.biopha.2017.10.090

[40] Jiang ZR , Gong J , Zhang ZN , Qiao Z . Influence of acid and bile acid on ERK activity, PPARgamma expression and cell proliferation in normal human esophageal epithelial cells. World J Gastroenterol. 2006;12:2445‐2449. 16688842 10.3748/wjg.v12.i15.2445 PMC4088087

[41] Gharibi B , Ghuman MS , Hughes FJ . Akt‐ and Erk‐mediated regulation of proliferation and differentiation during PDGFRβ‐induced MSC self‐renewal. J Cell Mol Med. 2012;16:2789‐2801. 22805337 10.1111/j.1582-4934.2012.01602.x PMC4118247

[42] Oak NR , Gumucio JP , Flood MD , et al. Inhibition of 5‐LOX, COX‐1, and COX‐2 increases tendon healing and reduces muscle fibrosis and lipid accumulation after rotator cuff repair. Am J Sports Med. 2014;42:2860‐2868. 25245131 10.1177/0363546514549943 PMC4246014

[43] Lin YM , Velmurugan BK , Yeh YL , et al. Activation of estrogen receptors with E2 downregulates peroxisome proliferator‐activated receptor gamma in hepatocellular carcinoma. Oncol Rep. 2013;30:3027‐3031. 24126791 10.3892/or.2013.2793

[44] Foryst‐Ludwig A , Clemenz M , Hohmann S , et al. Metabolic actions of estrogen receptor beta (ERbeta) are mediated by a negative cross‐talk with PPARgamma. PLoS Genet. 2008;4:e1000108. 18584035 10.1371/journal.pgen.1000108 PMC2432036

[45] Chu R , van Hasselt A , Vlantis AC , et al. The cross‐talk between estrogen receptor and peroxisome proliferator‐activated receptor gamma in thyroid cancer. Cancer. 2014;120:142‐153. 24114184 10.1002/cncr.28383

[46] Wang X , Liu J , Long Z , et al. Effect of diosgenin on metabolic dysfunction: Role of ERbeta in the regulation of PPARgamma. Toxicol Appl Pharmacol. 2015;289:286‐296. 26408789 10.1016/j.taap.2015.09.015

[47] Jeong S , Yoon M . 17beta‐Estradiol inhibition of PPARgamma‐induced adipogenesis and adipocyte‐specific gene expression. Acta Pharmacol Sin. 2011;32:230‐238. 21293475 10.1038/aps.2010.198 PMC4009938

[48] Tan Q , Lui PP , Rui YF , Wong YM . Comparison of potentials of stem cells isolated from tendon and bone marrow for musculoskeletal tissue engineering. Tissue Eng Part A. 2012;18:840‐851. 22011320 10.1089/ten.tea.2011.0362 PMC3313612

[49] Zhang J , Wang JH . Characterization of differential properties of rabbit tendon stem cells and tenocytes. BMC Musculoskelet Disord. 2010;11:10. 20082706 10.1186/1471-2474-11-10 PMC2822826

