# Supplementary material for: Absence of estrogen receptor beta leads to abnormal adipogenesis during early tendon healing by an up‐regulation of PPARγ signalling
Source: J Cell Mol Med. 2019 Sep 2;23(11):7406–16. doi: 10.1111/jcmm.14604 (PMC6815835; doi:10.1111/jcmm.14604)
Supplement: Supplementary file 1 [file JCMM-23-7406-s001.docx]

**Supplementary Fig. 1. A** Statistical analysis of CD34 positive cells in scar tissues revealed a significantly increase in ERβ^-/-^ mice compared with WT controls. **B** Statistical analysis of DAPI positive cells in scar tissues revealed a significantly decrease in ERβ^-/-^ mice compared with WT controls. Data are represented as mean±s.e.m (n=5), **p<0.01, ***p<0.001.

**Supplementary Fig. 2 A** Negative control. **B** CD18 negative. **C** CD34 negative. **D** CD44 positive. **E** D90 positive. Scale Bars: 100μm.

**Supplementary Fig. 3** Different estrogen receptors mRNA expression with different concentration of LY3201 of TDSCs. **Black column:** control, **Red column:** LY3201 10^-9^, **Blue column:** LY3201 10^-7^, **Green column:** LY3201 10^-5^.

**Supplementary Fig. 4 A** Immunofluorescence cytochemistry of ER-β of normal TDSCs. **B** Immunofluorescence cytochemistry of ERβ TDSCs treated with LY3201. **C** Immunofluorescence cytochemistry of ERβ of TDSCs treated with LY3201 and ROSI. Scale Bars: 100μm.

**Supplementary Fig. 5 A** Representative immunoblots of VEGFA and VEGFR. **B** Statistic analysis of VEGFA. **C** Statistic analysis of VEGFR. Data are represented as mean ± s.e.m (n=5), **p<0.01.

**Supplementary Fig. 6 A** Representative immunoblots of Phos-PTEN, PTEN, Phos-AKT, AKT, p53, Phos-ERK, ERK. **B** Statistic analysis of Phos-PTEN. **C** Statistic analysis of PTEN. **D** Statistic analysis of Phos-AKT. **E** Statistic analysis of AKT. **F** Statistic analysis of p53. **G** Statistic analysis of Phos-ERK. **H** Statistic analysis of Phos-ERK. Data are represented as mean ± s.e.m (n=5), **p<0.01, ***p<0.001.

**Supplementary Table S1** Histological scoring system^1^

**Supplementary Table S2 qt-PCR Primers**
